# Facile construction of manganese-based contrast agent with high *T*_1_ relaxivity for magnetic resonance imaging via flash technology-based self-assembly

**DOI:** 10.1093/rb/rbaf009

**Published:** 2025-03-11

**Authors:** Chunwei Wu, Jie Zhong, Jianing Li, Yande Luo, Junyao Wang, Xiaodie Zeng, Jiaji Mao, Jianping Lu, Junyao Xu, Changqiang Wu, Zhiyong Wang

**Affiliations:** School of Materials Science and Engineering, Center for Functional Biomaterials, Key Laboratory for Polymeric Composite and Functional Materials of Ministry of Education, Sun Yat-sen University, Guangzhou 510275, P. R. China; Medical Imaging Key Laboratory of Sichuan Province and School of Medical Imaging, North Sichuan Medical College, Nanchong 637000, P. R. China; Department of Radiology, Guangdong Provincial Key Laboratory of Malignant Tumor Epigenetics and Gene Regulation, Sun Yat-Sen Memorial Hospital, Sun Yat-Sen University, Guangzhou 510120, P. R. China; School of Biomedical Engineering, Guangzhou Medical University, Guangzhou 511495, P. R. China; School of Materials Science and Engineering, Center for Functional Biomaterials, Key Laboratory for Polymeric Composite and Functional Materials of Ministry of Education, Sun Yat-sen University, Guangzhou 510275, P. R. China; School of Materials Science and Engineering, Center for Functional Biomaterials, Key Laboratory for Polymeric Composite and Functional Materials of Ministry of Education, Sun Yat-sen University, Guangzhou 510275, P. R. China; Department of Radiology, Guangdong Provincial Key Laboratory of Malignant Tumor Epigenetics and Gene Regulation, Sun Yat-Sen Memorial Hospital, Sun Yat-Sen University, Guangzhou 510120, P. R. China; School of Materials Science and Engineering, Center for Functional Biomaterials, Key Laboratory for Polymeric Composite and Functional Materials of Ministry of Education, Sun Yat-sen University, Guangzhou 510275, P. R. China; Department of Hepatobiliary Surgery, The Third Affiliated Hospital, Guangzhou Medical University, Guangzhou 510150, P. R. China; Medical Imaging Key Laboratory of Sichuan Province and School of Medical Imaging, North Sichuan Medical College, Nanchong 637000, P. R. China; School of Materials Science and Engineering, Center for Functional Biomaterials, Key Laboratory for Polymeric Composite and Functional Materials of Ministry of Education, Sun Yat-sen University, Guangzhou 510275, P. R. China

**Keywords:** flash nanopreparation, magnetic resonance imaging, contrast agents, polyhenol, nanocomposite

## Abstract

To address the limitations of low relaxivity and physiological toxicity in commercial gadolinium-based contrast agents for magnetic resonance imaging (MRI), a novel manganese chelate macromolecular system was developed using a flash nanopreparation technique. Herein, the approach applying an instantaneous fluid device incorporated gallic acid, dopamine and Mn^2+^ to perform *in situ* polymerization of dopamine and covalent binding with albumin in a nanoconfined environment. This controllable self-assembly process characterized by its scalability and reproducibility was suitable for industrial-scale production. Under optimized flow rates and material ratios, the synthesized ultrasmall protein-based system, Mn-GA@BSA@DA, exhibited excellent aqueous dispersion with an average size of approximately 18 nm, allowing for long-term lyophilized powder storage. More importantly, the nanosystem demonstrated superior MRI-*T*_1_ relaxivity, significantly surpassing that of clinical gadopentetate dimeglumine, with a high value around 18.5 mM^−1^ s^−1^ and a low *r*_2_/*r*_1_ ratio (<5 at 3.0 T). Furthermore, this Mn-GA@BSA@DA contrast agent was endowed with tumor-targeting effects and a long MRI monitoring window period for the liver, gallbladder and renal tubules. The metal chelation within the nanoagent minimizes Mn^2+^ release; importantly, the antioxidant components, gallic acid and dopamine, significantly inhibit the Fenton reaction-induced toxicity, enhancing biocompatibility. Therefore, this study presents a simple and scalable production technique for a kind of MRI-*T*_1_-weighted contrast agent with high relaxivity and biocompatibility, offering a promising alternative to commercial Gd chelates.

## Graphical abstract

**Figure:**
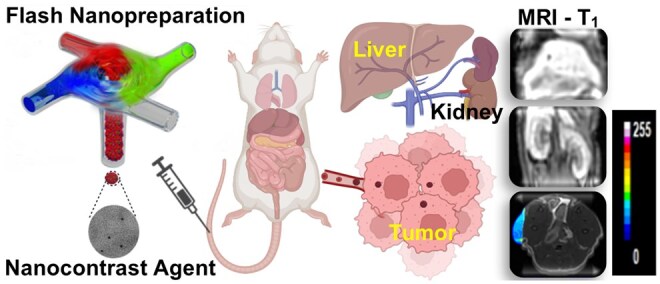

## Introduction

Magnetic resonance imaging (MRI) is a non-invasive medical imaging technique that utilizes radiofrequency pulses to excite hydrogen protons, generating signals from their transverse and longitudinal relaxation. These signals provide high-resolution biological information [1, 2]. However, the detection sensitivity of MRI is inherently limited by the low excitation efficiency of hydrogen protons due to molecular thermal motion, and the similar physiological microenvironment between initial lesions and normal tissues poses a challenge for accurate distinguishing [3].

MRI contrast agents, composed of magnetic substances, enhance diagnostic precision through altering the relaxation time of surrounding hydrogen protons, thus amplifying the contrast between normal and abnormal tissues [4]. These agents are categorized into *T*_2_ and *T*_1_ agents based on their physical properties. *T*_2_ agents, like superparamagnetic iron oxide nanoparticles, accelerate the proton’s transverse relaxation, leading to signal attenuation in *T*_2_-weighted images. However, this induces artifacts that could be confused with physiological signals like tissue hemorrhage or calcification and compromise diagnostic accuracy. Consequently, many commercial *T*_2_ contrast agents have been withdrawn from the market [5]. In contrast, *T*_1_ agents, primarily composed of paramagnetic metal ions and organic ligands, shorten the longitudinal relaxation time of hydrogen protons, increasing signal intensity in *T*_1_-weighted images without affecting the magnetic homogeneity or the anatomical background [6, 7]. Among clinical *T*_1_ agents, gadolinium (Gd)-based compounds, such as gadopentetate dimeglumine (Gd-DTPA), are widely applied due to their strong paramagnetic properties. However, the potential for Gd ions to detach from the chelating agents raises concerns about tissue accumulation and the risk of nephrogenic fibrosis and other diseases [8].

Recently, the development of non-gadolinium contrast agents has gained clinical attention. Manganese, as an essential metal element in human biology, emerges as a promising alternative due to its superior biocompatibility. Although manganese possesses five unpaired electrons, fewer than the seven in Gd, resulting in weaker paramagnetism, modifying manganese coordination complexes and encapsulating them with macromolecules like albumin could slow the rotational rate, significantly improving the imaging performance [9]. However, the intake of high concentrations of free Mn^2+^ can also cause damage to the human nervous system. Therefore, developing a manganese-based contrast agent with high relaxation rates, strong targeting capabilities and biological safety for *in vivo* MRI is still important. Currently, manganese-based contrast agents have been developed with various formulations, including manganese oxide nanocrystals and metal-organic frame nanoparticles [10, 11]. All of them are reported to exhibit high imaging relaxation, but metal nanocrystal particles, particularly manganese oxide contrast agents, have not yet been approved for clinical use, likely due to their metabolism and potential toxicological effects. Thus, preparing Mn-based contrast agents, especially using clinically approved molecules or ligands, show significant advantages in terms of safety, efficacy and biocompatibility. However, the synthesis steps for contrast agents are complex, and the modification processes need to be precisely controlled [11, 12]. Therefore, combining the advantages and disadvantages mentioned above, a simple preparation method, stable manganese chelation and high biosafety are the focus of our research on manganese-based contrast agents.

Gallic acid (GA) and dopamine (DA) are natural compounds with strong metal ions chelation abilities, as well as anti-inflammatory and antioxidant properties [13–15]. Utilizng these properties in contrast agent design is expected to repurpose “old” drug for new clinical translation. Beyond the coordination chemistry, effective surface modification also influences the pharmacokinetics of the contrast agents [16]. The non-toxic and non-immunogenic albumin is an ideal candidate for drug delivery systems. And its unique structure facilitates drug encapsulation through physical embedding or chemical bonding, while the negative charge and biocompatibility of albumin effectively prolong the circulation time. Herein, it becomes a challenge in the precise integration of albumin with the polyphenolic system to efficiently load Mn ions to develop manganese-based agents. Flash nanoprecipitation (FNP) is a continuous and rapid self-assembly method that transforms the preparation from thermodynamic to kinetic control. Utilizing a multi-inlet vortex mixer, FNP achieves producing nanoparticles with controllable aggregation through brief, high-speed turbulence, making it a promising method for the efficient preparation of nano-contrast agents [17, 18].

In this study, we employed the FNP technique to coordinate and self-assemble Mn^2+^, GA and DA molecules to prepare manganese-based MRI nano-contrast agents with high cost-effectiveness. Through the *in situ* polymerization of DA, this nanocomplex effectively conjugated bovine serum albumin (BSA). By predicting and adjusting synthesis parameters such as solvent flow rates and drug concentrations, we were able to regulate sizes and imaging function of contrast agents. The resulting manganese-based nanosystem demonstrated superior *T*_1_-MRI imaging performance compared to clinical Gd-based agents, with enhanced tumor-targeting diagnosis effect and long-term MRI monitoring capabilities. Notably, it enabled precise imaging of the liver, gallbladder and renal tubules, while exhibiting high biological safety *in vivo*.

## Materials and methods

### Materials

BSA, GA, manganese chloride and Nile red were purchased from Aladdin Biochemical Technology Co.; sodium hydroxide and hydrochloric acid DA were obtained from Macklin Biochemical Co.; IR-780 iodide was sourced from Merck; the CCK-8 kit was obtained from Biyuntian Biotechnology Co.; Dimethyl sulfoxide (DMSO) was purchased from Guanghua Technology Co; fetal bovine serum and penicillin-streptomycin solution were obtained from Thermo Fisher Scientific; and 1640 culture medium was sourced from Xiangpeng Biotechnology Co. Hoechst 33342 was purchased from Solarbio Technology Co. All reagents were used as received without further purification.

### Flash preparation of Mn-GA@BSA@DA contrast agent

Dissolve 20 mg of MnCl_2_, 50 mg of GA, 57 mg of BSA and 75 mg of DA separately in 20 ml of distilled water. Using FNP equipment, introduce the different solutions into four input ports at a flow rate of 5 ml/min. A light yellow-brown solution was obtained at the outlet, then it was stirred for an additional 4 h to ensure a complete reaction, resulting in a color change to deep black-brown. Mn-GA@BSA@DA contrast agents were obtained through dialysis for 24 h.To optimize the GA:DA feed ratio while keeping the concentration of GA solution of 2.5 mg/ml, varying molar ratios of GA:DA (3:1, 1:1, 1:2, 1:3) were employed. Similarly, when adjusting the feed ratio of DA:BSA in Mn-GA@BSA@DA contrast agent, the BSA solution was kept at 2.5 mg/ml, and different mass ratios of DA:BSA (0.5:1, 1:1, 1.5:1, 2:1) was applied; Furthermore, the flow rate parameters were adjusted at a different flow rate (2.5, 5,7.5, 10 ml/min). Finally, the synthesis of Mn-GA@BSA is regulated by replacing the DA solution with an equal amount of distilled water at the FNP input port, while all other reaction conditions were kept the same as those for the Mn-GA@BSA@DA contrast agent. In addition, Mn-GA@BSA and Mn-GA@BSA@DA contrast agents were mixed with ZnSO_4_ and (CH_3_COO)_2_Zn solution for 24 h, respectively, under different proportions (Mn:Zn = 1:3.6, 1:10, 1:40). The products were dialyzed for 24 h.

Two milligrams of Nile Red was dissolved in 40 ml of DMSO. The other components of the solution are the same as those used for the preparation of Mn-GA@BSA@DA contrast agent. Within FNP equipment, the Mn–GA mixed solution, BSA solution, DA solution and Nile Red solution were introduced into the four input ports at a flow rate of 5 ml/min. A light yellow-brown solution is obtained at the outlet, which is then stirred for an additional 4 h to ensure complete reaction. The solution is dialyzed for 24 h to obtain Mn-GA@BSA@DA@NileRed. For the synthesis of Mn-GA@BSA@NileRed, replace the DA solution with an equal volume of distilled water at the FNP input port, while keeping all other reaction steps the same as for Mn-GA@BSA@DA@NileRed. For the preparation of Mn-GA@BSA@DA@IR780 and Mn-GA@BSA@IR780, simply replace the Nile Red solution with IR780 iodide, while the remaining preparation steps remain the same.

### Structural characterization and imaging performance of Mn-GA@BSA@DA contrast agent

The TEM (Tecnai G2 Spirit T120 kV, Thermo, USA) was applied to characterize the structure of Mn-GA@BSA@DA contrast agent and the control group Mn-GA@BSA. A micro-confocal Raman spectrometer (inVia Qontor, RENISHAW, UK) was utilized with a 325 nm laser. The X-ray photoelectron spectrometeremployed for XPS assessment (ESCALAB 250, Thermo Fisher, USA). The nanoparticle size distribution and Zeta potential were analyzed via a nanoparticle size and potential analyzer (NanoBrook, Malvern, USA). The concentration of manganese in the Mn-GA@BSA and Mn-GA@BSA@DA contrast agent was determined with a plasma atomic emission spectrometer (ICAP Qc, Thermo, USA).

The relaxation times of nanoparticle aqueous solutions at different metal concentrations were obtained using MRI systems with magnetic fields of a clinical 3.0 T system (United Imaging, China). The relaxation time inverses (1/*T*_1_ or 1/*T*_2_, s^−1^) were fitted against manganese concentration (mM) to calculate the *T*_1_ or *T*_2_ relaxivity (*r*_1_ or *r*_2_). Additionally, MRI was performed on these solutions and ultrapure water at 3.0 T. Herein, the imaging parameters were as follows: For *T*_1_-weighted imaging (*T*_1_ WI), fast spin echo (FSE) sequence, TR = 300 ms, TE = 12 ms, FOV = 220 mm × 200 mm, slice thickness = 1 mm, flip angle = 135°; for *T*_2_-weighted imaging (*T*_2_ WI), the FSE sequence, TR = 4500 ms, TE = 200 ms, FOV = 220 mm × 200 mm, slice thickness = 2 mm, flip angle = 140°.

### Manganese ion release experiment

Seven hundred fifty microliters of Mn-GA@BSA and Mn-GA@BSA@DA contrast agent was placed in a dialysis membrane, which was then transferred into a 50 ml centrifuge tube containing 30 ml of release medium. The release medium consisted of buffer solutions at pH 7.4 and pH 6.4, with a sample-to-dialysis fluid volume ratio of 1:40. The dialysis apparatus was positioned vertically on a shaker, and at time points (0.5, 1, 2, 4, 6, 8, 24 h), 2 ml of the release medium was taken for concentration measurement, and an equal volume of medium was added back. The cumulative release rate of manganese ions was calculated using the following formula:


$$E_{r}=\frac{V_{e}\sum_{1}^{n-1}C_{i}+V_{0}C_{n}}{m_{Mn^{2+}}}\;$$

where E_r_ is the cumulative release amount, V_e_ is the replaced volume, V_0_ is the total volume of the release medium, C_i_ is the concentration of the release fluid at the ith sampling, m_Mn^2+^_ is the total mass of the drug carried by the nanoparticles; and n is the number of times the release medium is replaced (n = 3).

### Cytotoxicity of Mn-GA@BSA@DA contrast agent

In this research, the mouse embryonic fibroblast cell line (3T3), mouse neural stem cell line (C17.2) and mouse breast cancer cell line (4T1) were utilized and they were cultured with dulbecco's modified eagle medium (DMEM) high-glucose medium and RPMI 1640 medium, respectively. All cell cultures were kept in a controlled incubator environment within 37°C and 5% CO_2_. The 3T3, C17.2 and 4T1 cell lines were employed to investigate the cytotoxic effect of Mn-GA@BSA@DA contrast agent. The three types of cells were plated in a 96-well format with a density of 6 × 10^4^. Cells were incubated for 24 h with a culture medium containing varying concentrations of Gd-DTPA, Mn-GA@BSA or Mn-GA@BSA@DA contrast agent ([Mn/Gd]: 0, 1, 3, 5, 7, 9 μg/ml), while PBS was used as a negative control. Subsequently, CCK-8 reagent was added into the culture medium according to the protocol. And the absorbance was measured with a multifunctional microplate reader (Synergy2 Gen5, Biotek, America) to assess cell viability following the formula:


$$Cell\;viability\;(\%)=\frac{450\;nm\;absorbance\;of\;sample}{450\;nm\;absorbance\;of\;control}\times 100\%$$

### Hemocompatibility detection of contrast agents

Blood samples were collected from the orbital cavities of Balb/c mice and rinsed with saline solution. Subsequently, the blood underwent centrifugation at 1800 rpm for a duration of 15 min, after which the supernatant was discarded; and this process was conducted 3 times. An appropriate volume of saline was then introduced to the red blood cells to create a suspension. Different concentrations of Mn-GA@BSA and Mn-GA@BSA@DA contrast agent solutions were prepared with [Mn]: 0, 0.5, 1, 3, 5, 20 µg/ml. A 100-µl red blood cell suspension was combined with 900 µl of the contrast agent solution. Additionally, the PBS and pure water were served as negative and positive controls, respectively. The samples were gently mixed on a shaker for 60 min, followed by centrifugation at 3000 rpm for 15 min at 4°C. Subsequently, 100 µl of the supernatant was transferred into a 96-well plate, and absorbance was assessed at 540 nm, enabling the calculation of the hemolysis rate. The formula for hemolysis rate is:


$$Hemocompatibility\;(\%)=\frac{Sample\;absorbance\;-Negative\;control\;absorbance}{Positive\;control\;absorbance\;-Negative\;control\;absorbance}\times 100\%$$

### Cellular uptake of contrast agent

4T1 cells were utilized to investigate the cellular uptake of Mn-GA@BSA and Mn-GA@BSA@DA particles. The cells at a density of 6 × 10^4^ were cultured in a 24-well plate. After 24 h, the media were supplemented with Mn-GA@BSA@Nile Red or Mn-GA@BSA@DA@Nile Red at an Mn concentration of 5 µg/ml. Incubation times were established at 0.25, 1, 4 and 24 h (n=3), respectively. After then, the cells were washed with PBS and stained with Hoechst 33342. The uptake of the nanoparticles at various time points was assessed using an inverted fluorescence microscope (MF52-N, Mshot, China). Subsequently, the cells were digested, and the quantity of Nile Red dye uptake was analyzed using the PI channel on a flow cytometer (NovoCyte 3000, Agilent, America). The cellular uptake was also evaluated using confocal laser scanning microscopy (Nikon C2).

### Single-cell metal content determination

Cell uptake of Gd-DTPA, Mn-GA@BSA and Mn-GA@BSA@DA granules was observed in 4T1 cells. Cells with a density of 6 × 10^4^ were cultured with different samples in 24-well plates for 0.25, 4 and 24 h, respectively (n=3). The cells were washed with PBS, collected and counted. The metal (Mn/Gd) content in individual cells was determined using Inductively Coupled Plasma (Optima 8300, PerkinElmer, America).

### Cellular magnetic resonance experiments

Cellular magnetic resonance experiments of Mn-GA@BSA and Mn-GA@BSA@DA contrast agents were studied using 4T1 cells. The Gd-DTPA, Mn-GA@BSA and Mn-GA@BSA@DA contrast agents with 5 µg/ml Gd and Mn concentrations were added into the medium, respectively. After incubation for 24 h, we washed the cells with PBS. After digestion, the cells were divided into 5, 25, 50, 100, 175, 2 million cell concentrations and then added 1% agarose gel (n=3). MRI of cell gels was performed using the clinical 3.0T MRI system (United Imaging, China). The imaging parameters were set as follows: *T*_1_WI, an FSE sequence was used with parameters: TR = 200 ms, TE = 4 ms, FOV = 100 mm × 100 mm, slice thickness = 1.4 mm, flip angle = 70°.

### *In vivo* pharmacokinetic experiments and biosafety analysis

All animal procedures were carried out following the care and use guidelines set forth by the Animal Experiment Center of Sun Yat-Sen University, and the Ethics Committee of the same center approved the experiments (ethics number 2024001116).

Tail vein injections of IR780, Mn-GA@BSA@IR780 and Mn-GA@BSA@DA@IR780, respectively, were administered to Balb/c female mice, at a drug concentration of 5 µg/ml for IR780. Near-infrared *in vivo* imaging was performed using an *In-Vivo* Master imaging system with an 808 nm laser at different time points (1, 3, 5, 7, 11, 24, 31, 48 h) to assess fluorescence signal in the dorsal and tumor regions of the mice. Fluorescence information from the tumor site on the right leg and the corresponding site on the left leg was analyzed using ImageJ software following the formula:


$$SNR=\frac{Fluorescence\;value\;of\;the\;tumor\;site}{Fluorescence\;value\;of\;the\;corresponding\;site\;on\;the\;left\;leg},n=3$$

The blood samples were collected from orbital cavities of mice and centrifuged at 1800 rpm to obtain the supernatant for routine hematological analyses. The mice were subsequently euthanized, and the organs including heart, liver, spleen, lungs and kidneys along with tumors were excised for fluorescence imaging. Subsequently, the extracted organs were then prepared for tissue sectioning, stained with hematoxylin and eosin(HE), and subjected to histopathological analysis.

### *In vivo* magnetic resonance experiments

A clinical 3.0 T MRI system (United Imaging, China) was used to perform MRI on both normal female mice and mice bearing 4T1 tumor. Following the intravenous injection of the materials via the tail vein, *T*_1_-weighted imaging was conducted at different time points, capturing both whole-body coronal and tumor axial views. The imaging parameters were set as follows: for whole-body coronal *T*_1_WI, an FSE sequence was used with parameters: TR = 9 ms, TE = 4 ms, FOV = 120 mm × 80 mm, slice thickness = 0.5 mm, flip angle = 10°. For tumor axial *T*_1_WI, the FSE sequence parameters were: TR = 294 ms, TE = 13 ms, slice thickness = 1 mm, FOV = 60 mm × 60 mm, flip angle = 150°.

## Results and discussion

### Flash preparation of Mn-GA@BSA@DA contrast agent

**Figure 1.**
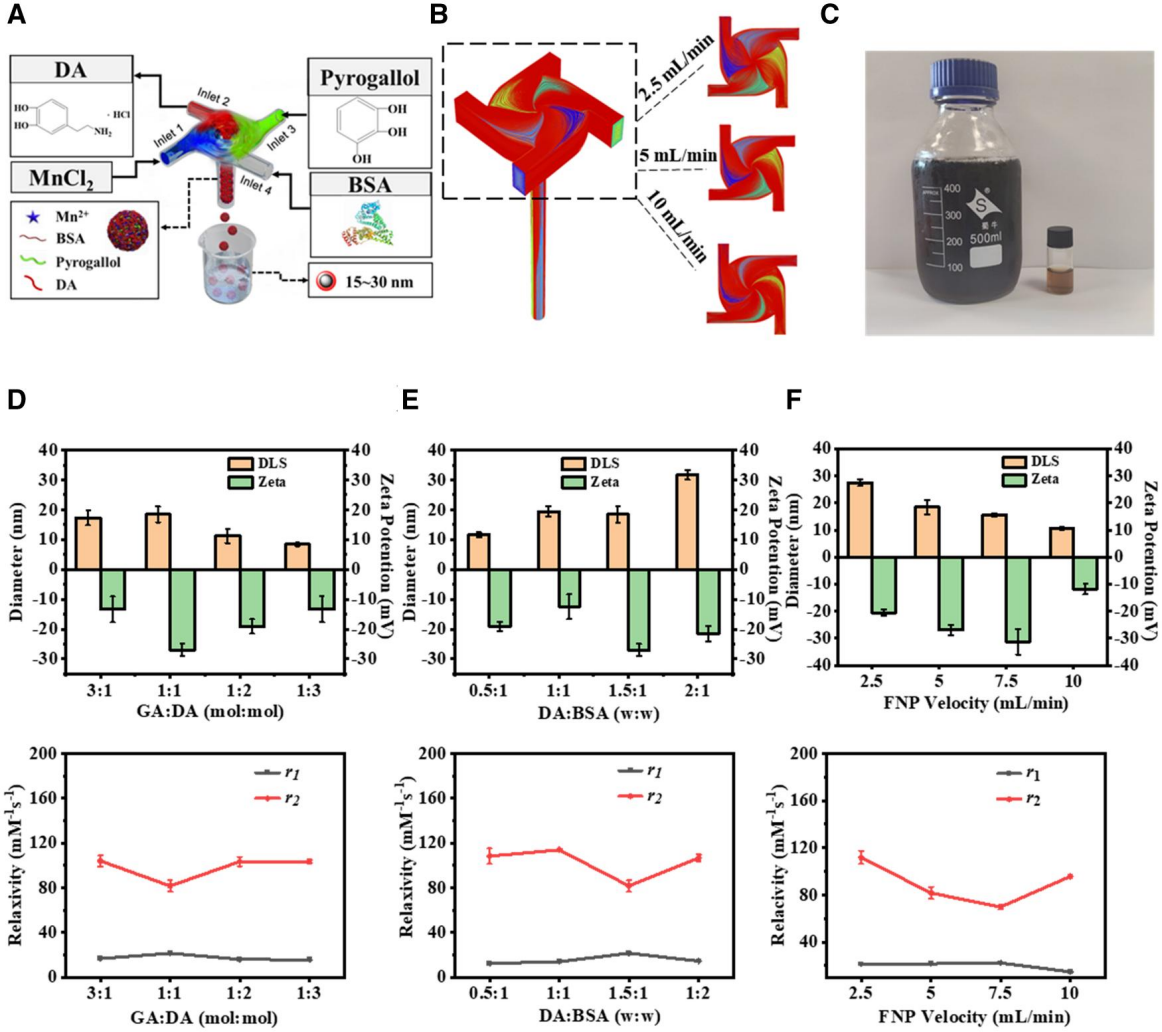
(**A**) Schematic diagram of FNP preparation of Mn-GA@BSA@DA contrast agent. (**B**) Fluid trajectory simulation of FNP at varying flow rates. (**C**) Batch preparation images of Mn-GA@BSA@DA samples using FNP technique. (**D**–**F**) Analysis of particle size, zeta potential and MRI- *T*_1_ and *T*_2_ relaxivity of nano-contrast agent at different GA:DA ratios, DA:BSA ratios and flow rates, respectively (*n*=3).

FNP is an efficient technique for nanoparticle preparation, using a multi-channel vortex system to achieve controllable mixing of solvents and materials. This method is advantageous for its mechanical automation, high reproducibility and minimal human intervention [19]. In this study, the FNP technology was employed to create Mn-based contrast agent by coordinating Mn ions with polyphenolic compounds, inducing DA self-polymerization and conjugating with albumin in the nanoformulation, which enabled the rapid self-assembly of multicomponent within sub-millisecond timescales (Figure 1A).

To optimize this synthesis process, we established a structural model to simulate the internal flow dynamic of the FNP apparatus, visualizing the preparation process (Figure 1B). Employing a structured grid finite element method, we set several parameters such as flow rates, feed ratios and the solvent’s density and viscosity. The simulation results showed symmetrical fluid flow trajectories, indicating a uniform reaction environment within the FNP system. This allowed reactants to rapidly self-assemble into nanoparticles under controllable surface forces. Increasing the flow velocity enhances the surface forces exerted on the reactant, resulting in a reduced trajectory range of the fluid [20]. Practical experiments confirmed the scalability of this method, successfully producing both small (5 ml) and large (0.5 l) batches under 5 min (Figure 1C), highlighting its potential for meeting industrial production demands.

In the FNP preparation system, the uniform stress environment resulted in well-formed nanostructures with controllable particle size distribution, which could be adjusted through different feed ratios and flow rates [21, 22]. Increasing the DA proportion, gradually decreased the size of the nanoparticle (Figure 1D), suggesting enhanced the self-polymerization and cross-linking. Given the intended application of these nanocomposites as MRI contrast agents, it is essential to assess their imaging effect. And the relaxivity ratio (*r*_2_/*r*_1_), a critical parameter for *T*_1_ contrast agent, typically requires <20 to effectively enhance the longitudinal relaxation effect [23]. As shown in Figure 1D, the nanoparticles under varying GA ratios exhibited *r*_2_/*r*_1_ ratios below 20, with the optimal GA:DA ratio of 1:1 yielding an enhanced transverse-relaxation rate (*r*_1_) value of 21.61 (mM^−1^ s^−1^) and a minimum *r*_2_/*r*_1_ ratio of 3.78 under 1.5 T magnetic field environment, indicating superior *T*_1_ imaging performance. The strong chelation of Mn^2+^ by GA and DA, combined with albumin bonding, significantly increased nanoparticles hydrophilicity enhancing their interaction with water protons and improving *T*_1_ relaxation rates [24].

Additionally, albumin serves as a surface-coating macromolecule influencing both contrast agent system’s stability and MRI imaging. To optimize these factors, we maintained a fixed GA:DA ratio of 1:1 and then varied BSA content. In Figure 1E, the particle size was increased from 11.61 to 31.65 nm by reducing the BSA content. At a DA:BSA ratio of 1.5:1, a high negative zeta potential of the contrast agent was measured with −26.93 ± 2.06 mV, which could contribute to its excellent stability. Further observations revealed that the group with the lowest BSA content (2:1 ratio) exhibited some sedimentation during long-term storage (data not shown). More importantly, the albumin modification could slow down the rotational diffusion rate of the contrast and significantly enhance the relaxation rate [25]. At the DA:BSA ratio of 1.5:1 condition, it also demonstrated the best *T*_1_ imaging effect among the tested groups. Flow rate was another key factor. Higher rates resulted in shorter mixing times impacting the components binding within microenvironment [26]. In Figure 1F, the contrast agent constructed within a flow rate of 5 ml/min exhibited the best MRI-*T*_1_ imaging performance. Therefore, the parameters of GA:DA = 1:1, DA:BSA = 1.5:1 and a flow rate of 5 ml/min was selected for the following experiments.

### Structural characterization and imaging performance of Mn-GA@BSA@DA contrast agent

**Figure 2.**
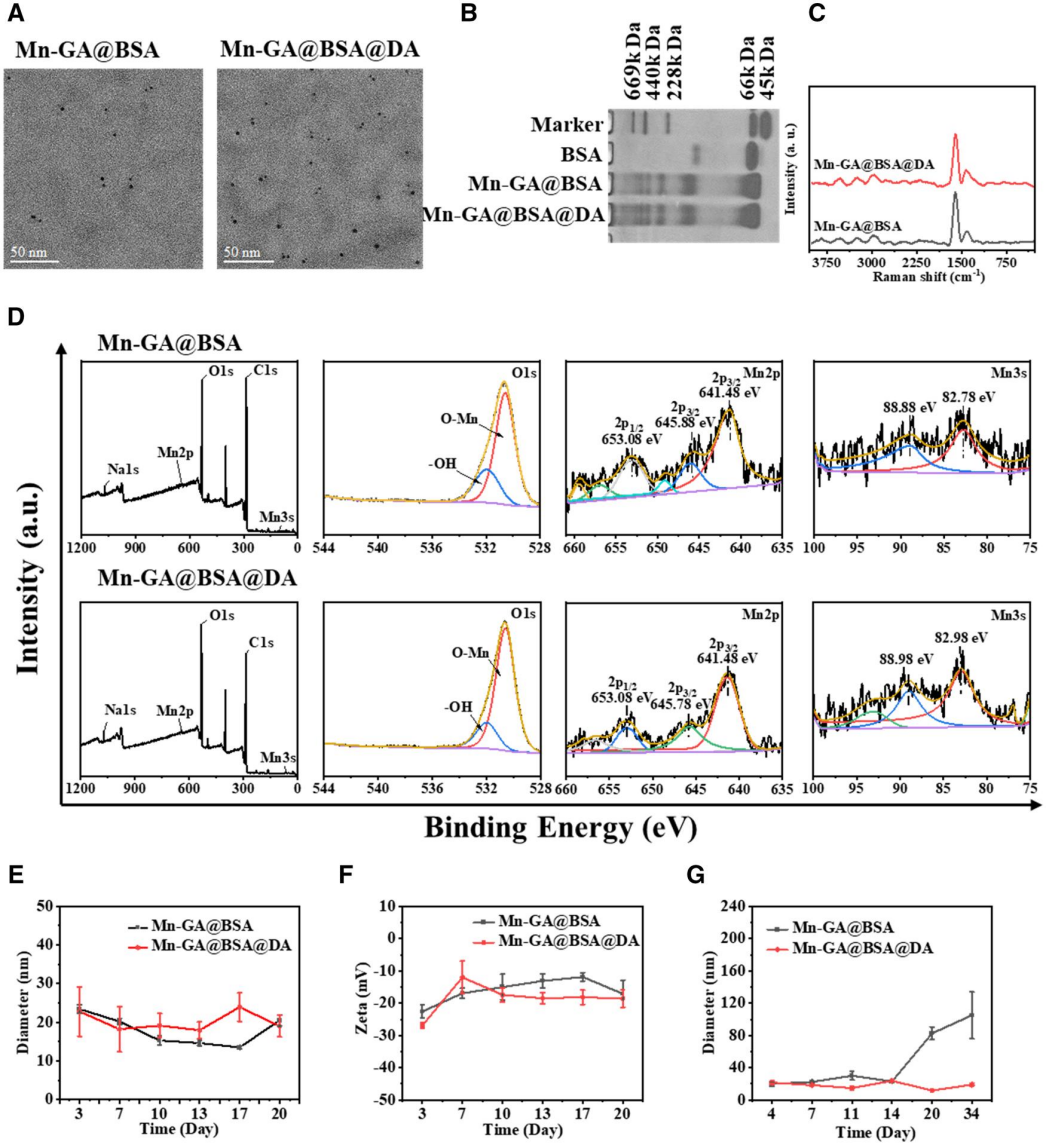
Structural characterization of Mn-GA@BSA@DA contrast agent. (**A**) TEM images of Mn-GA@BSA@DA agent and control Mn-GA@BSA nanoparticle, with a scale bar of 50 nm. (**B**) Non-denaturing polyacrylamide gel electrophoresis results. (**C**) Raman spectroscopy analysis. (**D**) X-ray photoelectron spectroscopy data. (**E** and **F**) The size and Zeta potential stability of nano-contrast agent through dynamic light scattering analysis over different times points (3, 7, 10, 13, 17, 20 days), respectively. (**G**) The stability analysis of nano-contrast agents in cell culture medium at 37°C over various durations (4, 7, 11, 14, 20, 34 days).

To assess the assembly structure of the contrast agent, we first characterized the nanoparticles’ morphology using transmission electron microscopy (TEM), with the Mn-GA@BSA without DA serving as the control group. As illustrated in Figure 2A, both Mn-GA@BSA and Mn-GA@BSA@DA exhibited uniform spherical shapes. The particle size distribution obtained from TEM showed around 3 nm (Figure S1A), which was smaller than that from dynamic light scattering. This difference was likely due to the fact that the protein shell of nano-contrast agent under TEM was difficult to distinguish and it was consistent with other literature reports [27, 28]. Non-denaturing polyacrylamide gel electrophoresis further confirmed the successful integration of BSA into nanostructure, as indicated by altered migration patterns and band tailing (Figure 2B).

We also analyzed the valence state of Mn element and coordination in the contrast agents using Raman spectroscopy and X-ray photoelectron spectroscopy (XPS). In Figure 2C, Raman spectra indicated that both Mn-GA@BSA@DA and the control Mn-GA@BSA group exhibited peaks at 1589.3 cm^−1^ (C=C) and 1411.04 cm^−1^(C–O). There were not any characteristic peaks of standard MnO (690, 634, 298, 197 cm^−1^) or Mn_3_O_4_ nanocrystals (647, 353, 308 cm^−1^) [29, 30]. It confirmed that Mn^2+^ did not undergo oxidation during the preparation process, and no by-products were generated. The XPS analysis revealed the valence states of Mn. And the O1s spectrum in Figure 2D displayed two peaks with a lower Binding Energy (BE) peak (530.58 eV) corresponding to O–Mn bonding, and a higher peak (532.03 eV) corresponding to –OH functional groups [31]. The Mn2p spectrum showed two distinct peaks around 641.48 and 653.08 eV, with an energy gap (Eg) of 11.6 eV, indicating the presence of Mn^2+^ [32]. Additionally, the Mn3s spectrum revealed peaks at 82.88 and 88.98 eV, with an energy gap 6.1 eV, consistent with Mn–O bonding [33]. Therefore, the successful attachment of Mn^2+^ components in the contrast agent was confirmed.

The stability of the nanostructure was further investigated in both MilliQ water and cell culture medium. In Figure 2E and F, dynamic light scattering (DLS) characterization over 20 days revealed that both tested and control nanosystem maintained a stable size around 20 nm and zeta potentials close to –20 mV in MilliQ water. In serum and salt-rich medium over 34 days, there was minimal change in the size of Mn-GA@BSA@DA agent; in contrast, the control Mn-GA@BSA showed significant size increases (104.94 ± 29.07 nm in Figure 2G). Similarly, Mn-GA@BSA@DA nanoparticles could maintain constant particle size within different proportions of Zn^2+^ solution under pH 6.4 (Table 1), which indicated that the *in situ* polymerization of DA in Mn-GA@BSA@DA nanoparticles effectively maintained structural stability. In addition, the lyophilized Mn-GA@BSA@DA contrast agents demonstrated rapid re-dissolution with a minimal size variation (Figure S1B). This indicated that Mn-GA@BSA@DA contrast agents could be stored for extended periods, with their high hydrophilicity and excellent stability.

**Table 1.** Particle sizes of Mn-GA@BSA and Mn-GA@BSA@DA contrast agents in ZnSO_4_ and (CH_3_COO)_2_Zn solutions with Mn:Zn ratios of 1:3.6, 1:10 and 1:40, respectively

Diameter (nm)	Mn:Zn = 1:3.6	Mn:Zn = 1:10	Mn:Zn = 1:40
Mn-GA@BSA+ZnSO_4_	34.03 ± 0.24	28.07 ± 2.75	Have precipitated
Mn-GA@BSA@DA+ZnSO_4_	19.55 ± 1.12	16.76 ± 3.50	12.56 ± 0.56
Mn-GA@BSA+(CH_3_COO)_2_Zn	24.73 ± 3.60	25.91 ± 6.24	Have precipitated
Mn-GA@BSA@DA+(CH_3_COO)_2_Zn	18.97 ± 1.86	23.00 ± 2.49	21.46 ± 2.15

**Figure 3.**
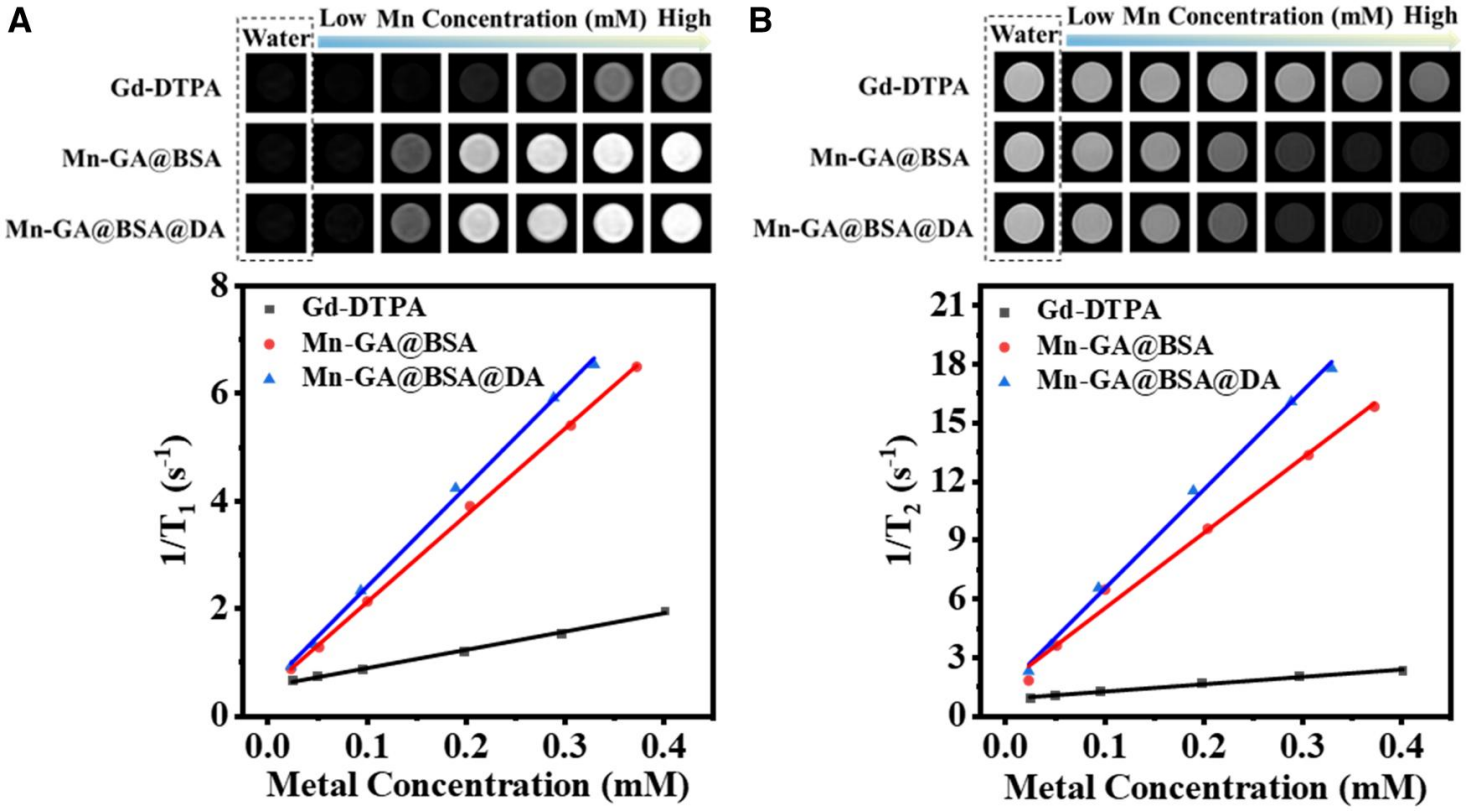
Magnetic resonance imaging analysis of Gd-DTPA, Mn-GA@BSA, and Mn-GA@BSA@DA contrast agents. (**A**) MRI *T*_1_-weighted images and corresponding relaxation curves; (**B**) MRI *T*_2_-weighted images and corresponding relaxation curves.

To assess the MRI effect of the Mn-based agent, a clinical 3.0 T MRI imaging system was employed. Herein, the commercial Gd-DTPA agents served as the positive control. As shown in Figure 3A and B, increasing Mn concentration resulted in progressively brighter *T*_1_ images across all samples. At equivalent metal concentration, Mn-GA@BSA@DA exhibited a superior *T*_1_ imaging contrast, significantly outperforming Gd-DTPA. And the *T*_1_ relaxivity of Mn-GA@BSA@DA particles was 18.5 mM^−1 ^s^−1^, which was 5.4 times higher than that of Gd-DTPA at 3.0 T clinical scanner, while its *T*_2_ relaxivity was 50.5 mM^−1 ^s^−1^, 13.4 times that of Gd-DTPA, highlighting the excellent imaging capability of Mn-GA@BSA@DA agent. Compared with small-molecule Gd-DTPA, the Mn-GA@BSA@DA and Mn-GA@BSA nano-contrast agent are metal coordination polymers with significantly higher molecular weight (>5000 Da) than that of Gd-DTPA (938 Da) [34–38]. Thus, the elevated *T*_1_ relaxation rate of nano-contrast agents is likely attributed to the increased molecular weight and volume of space, which extends the rotational correlation time [39, 40].

### Cytotoxicity and cellular imaging of Mn-GA@BSA@DA contrast agent

**Figure 4.**
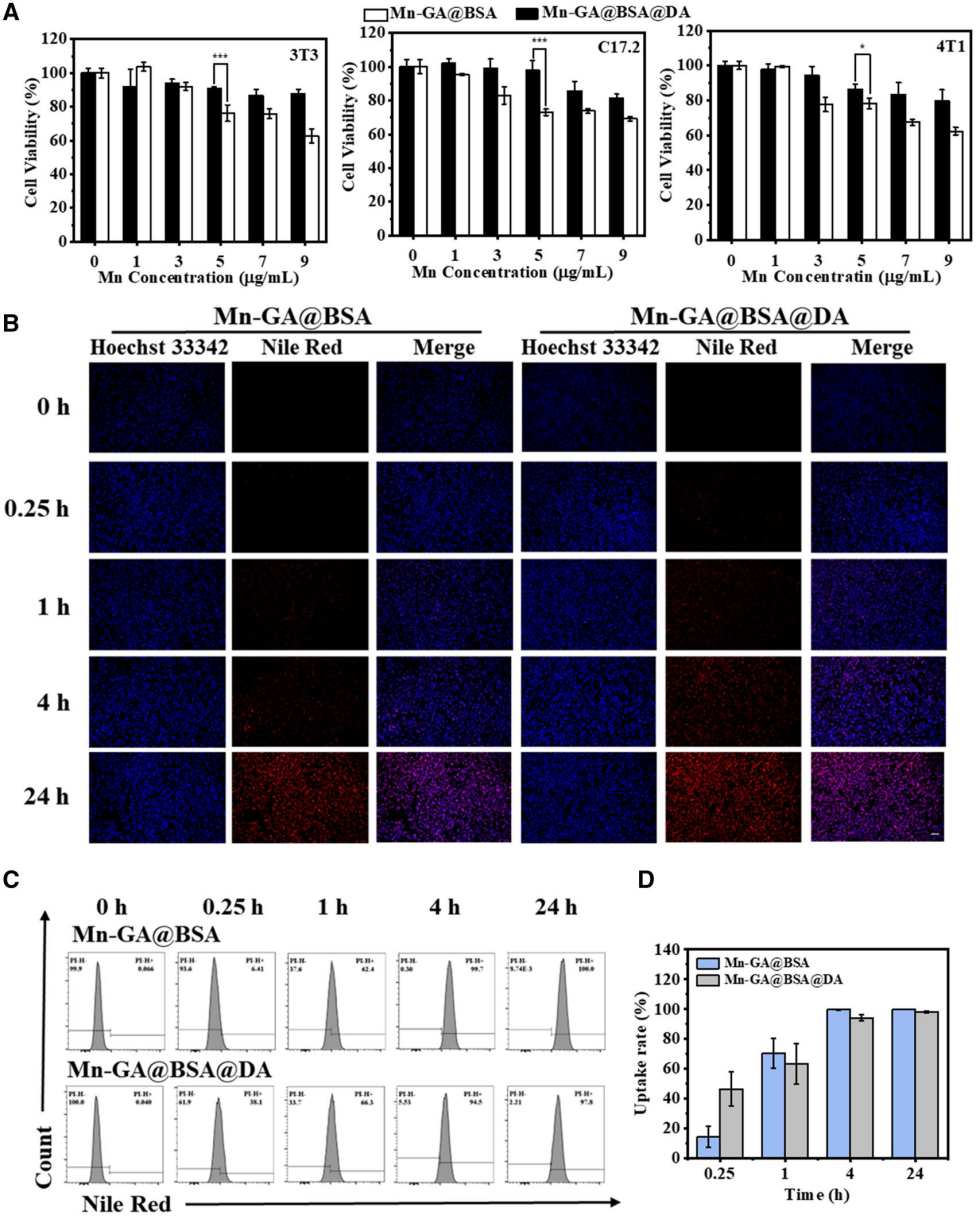
(**A**) Cytotoxicity assessment of various contrast agents on 3T3, C17.2 and 4T1 cells measured by the CCK-8 assay (*n*=3). (**B**) Fluorescent images of 4T1 cells incubated with contrast agent for different times (0, 0.25, 1, 4, 24 h), scale bar = 50 μm; (**C** and **D**) Time-dependent cell uptake of different contrast agents visualized under the PI channel using flow cytometry; statistical analysis of flow cytometry data (*n*=3).

Having established effective MRI-*T*_1_ contrast imaging capabilities of Mn-GA@BSA@DA agents, we proceeded to evaluate the cytocompatibility to confirm their potential for biological applications. In this study, the CCK-8 assay was applied to measure the cell viability under varying manganese concentrations on embryonic fibroblasts (3T3), neural stem cells (C17.2) and breast cancer cells (4T1). As shown in Figure 4A, at Mn-GA@BSA@DA concentrations ranging from 1 to 9 [Mn] µg/ml, the viability of all three cell types remained above 80%, even at a high concentration of 9 μg/ml [Mn] for 24 h, indicating low cytotoxicity. In contrast, the control group Mn-GA@BSA showed a reduction in cell viability to around 60% at 9 µg/ml. Further assessments of the individual components, GA and Mn^2+^, revealed that GA was largely no-toxic, maintaining above 90% cell viability even at 17.5 µg/ml (Figure S2A). However, the toxicity of MnCl_2_ increased with the concentration (Figure S2B). And these results highlighted the role of chelation in mitigating manganese-related toxicity within nanosystems. In addition, the cellular activity from the clinical Gd-DTPA group was also reduced to 80% under 9 μg/mL [Gd] (Figure S2C), but there was low uptake of Gd-DTPA by cells (Figure S7). The intracellular metal concentration detection results (Figure S7) showed that at 24 h, the intracellular Gd^3+^ content in the Gd-DTPA group was only 0.75 pg metal ions/cell, while the intracellular Mn^2+^ content in the Mn-GA@BSA and Mn-GA@BSA@DA groups could increase to 5.32 and 5.16 pg per cell, respectively. This result was consistent with the previous report that cells have taken up less Gd-DTPA, but it caused a high toxic effect on cell activity [41]. In contrast, the endocytosis of Mn-based nanoparticles was significantly higher than that of Gd-DTPA, further supporting the superior biocompatibility of Mn-based nanomaterials.

To investigate whether cytotoxicity was linked to the release of Mn ions, drug release experiments were conducted under different pH conditions (Figure S3A and B). In a pH 7.4 environment, both two types of particles released <1% of manganese. However, under acidic conditions (pH 6.4), the control Mn-GA@BSA particles released around 11% of Mn within 24 h, whereas Mn-GA@BSA@DA agents showed only around 7.4%, and only about 8% was released even at 40 times the concentration of Zn^2+^ solution, suggesting that the toxicity of the nanosystem was due to the released free Mn^2+^, which might induce the Fenton reaction to produce highly toxic hydroxyl radicals and led to cellular dysfunction. Further exploration of the toxic catalytic behavior of the Mn-based contrast agent using H_2_O_2_ as a substrate confirmed this hypothesis.

In Figure S4, The MnCl_2_ as the positive control group exhibited a distinct blue color and a strong absorption peak at 652 nm due to the generation of hydroxyl radicals that oxidized 3,3,5,5-tetramethylbenzidine (TMB) to form the oxTMB complex. Previous reports indicate that phenolic hydroxyl groups serve as effective nucleophiles [42]. In the nanocomposite, the hydroxyl structure could prevent the interaction between hydroxyl radicals and hydrogen peroxide with the phenolic-metal complex, thereby reducing the metal ions oxidization and inhibiting the Fenton reaction [43–46]. Consistent with these findings, our experiments showed that the Mn-GA@BSA@DA group effectively suppressed the Fenton reaction and reduced the generation of toxic radicals by stabilizing the metal ions in the contrast agent, which enhanced the nanoparticle’s biosafety for medical applications.

In clinical applications, contrast agents direct contact with the blood, making blood compatibility another important indicator. Hemolysis assays revealed that the positive control (water) rapidly caused red blood cell membrane rupture, leading to the release of hemoglobin and heme, which led to light absorption at 540 nm (Figure S5A and B). In contrast, at a concentration of 20 µg/ml, both Mn-GA@BSA@DA and the control Mn-GA@BSA exhibited hemolysis rates below 5%, with no significant change in the red blood cell morphology. These results underscore the safety of Mn-based contrast agent for *in vivo* administration. Based on cytotoxicity and hemolysis test, a Mn^2+^ concentration of 5 µg/ml was deemed safe, and subsequent experiments were conducted using this concentration.

Nile red is a fluorescent dye and has been widely used for nanoparticle tracking in cell [47]. To investigate the cellular metabolism, we labeled the contrast agent with Nile Red dye and used Hoechst 33342 to stain the cell nuclei [48, 49]. Some studies have shown that the Nile red in the nanoparticles would react with the intracellular lipids to produce fluorescence after entering the cell, and then, indicating the uptake of the substance by the cell [50, 51]. Through the confocal scanning laser microscope and fluorescence microscope analysis, the red fluorescence dots in cytoplasm could be observed in almost all the nano-contrast agent labeled cells at 4 h (Figure S6 and Figure 4B), further confirming that nanoparticles could be effectively internalized by cells. The flow cytometry also confirmed that the exceeded 95% of cells were labeled by nanoparticles, nearing saturation (Figure 4C and D).

**Figure 5.**
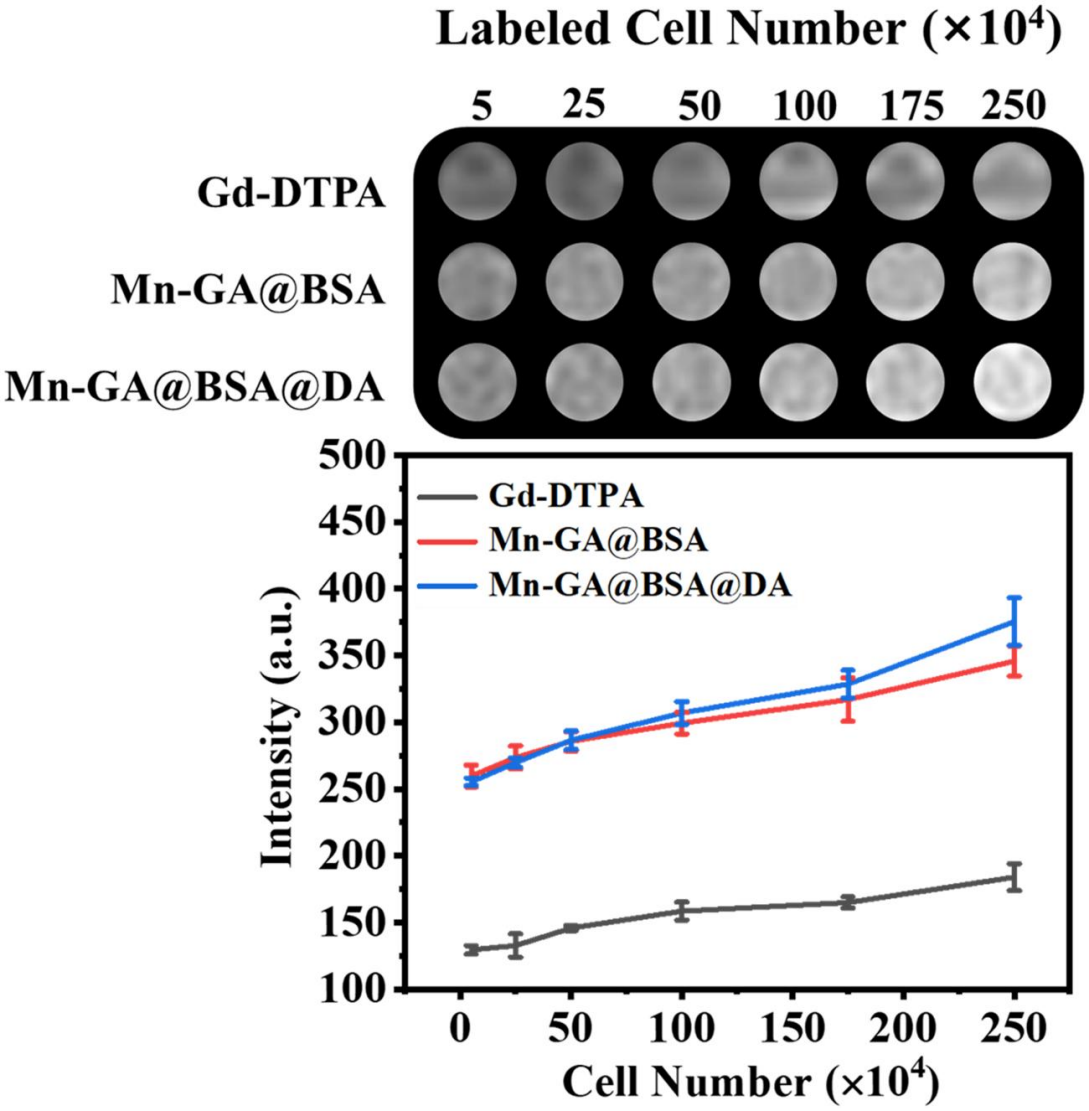
MRI-*T_1_*-weighted imaging of the labeled 4T1 cells and the relationship between signal intensity and cell count (*n*=3).

Following cellular endocytosis, the contrast agent’s magnetic properties shorten the longitudinal relaxation time of hydrogen protons in the cellular environment and enable the effective MRI identification of cells. After 24 h co-incubation of nanoparticles with 4T1 cells, *T*_1_ time changes were evaluated at varying cell counts using a clinical 3.0 T MRI system. As shown in Figure 5, the MRI-*T*_1_ signal intensity from both three samples increased with cell count, resulting in progressively brighter images. Further analysis of signal intensity revealed that the signal value for the Mn-GA@BSA@DA group increased from 255.34 to 375.43 with the cells’ number changed from 50 000 to 2 500 000. On the contrary, the signal value of the Gd-DTPA group with a high cell count (25 × 10^5^) was only 183.98, which was even lower than that of Mn-GA@BSA@DA contrast agent (255.34) at a cell count 5 × 10^4^. In Figure S7, the endocytosis effect of Gd-DTPA cells was significantly lower than that of nano-contrast agent groups, which was one of the reasons for the better MRI performance of labeled cells in Mn-GA@BSA@DA and Mn-GA@BSA groups. Compared to the Mn-GA@BSA control group, the Mn-GA@BSA@DA test one exhibited superior *T*_1_ contrast imaging, indicating that Mn-GA@BSA@DA had higher sensitivity, which laid a foundation for the *in vivo* experiments.

### Metabolic behavior of Mn-GA@BSA@DA nanoparticles *in vivo*

Magnetic resonance contrast agents enhance imaging by exploiting differences in hemodynamics and vascular permeability between diseased and normal tissues [52]. Therefore, understanding the metabolic behavior of these contrast agents is critical. In this study, near-infrared II fluorescence imaging, known for its real-time observation capabilities, high tissue penetration and sensitivity, was applied to comprehensively investigate the metabolism of Mn-based contrast agents labeled with IR-780 dye in a 4T1 subcutaneous tumor mouse model.

**Figure 6.**
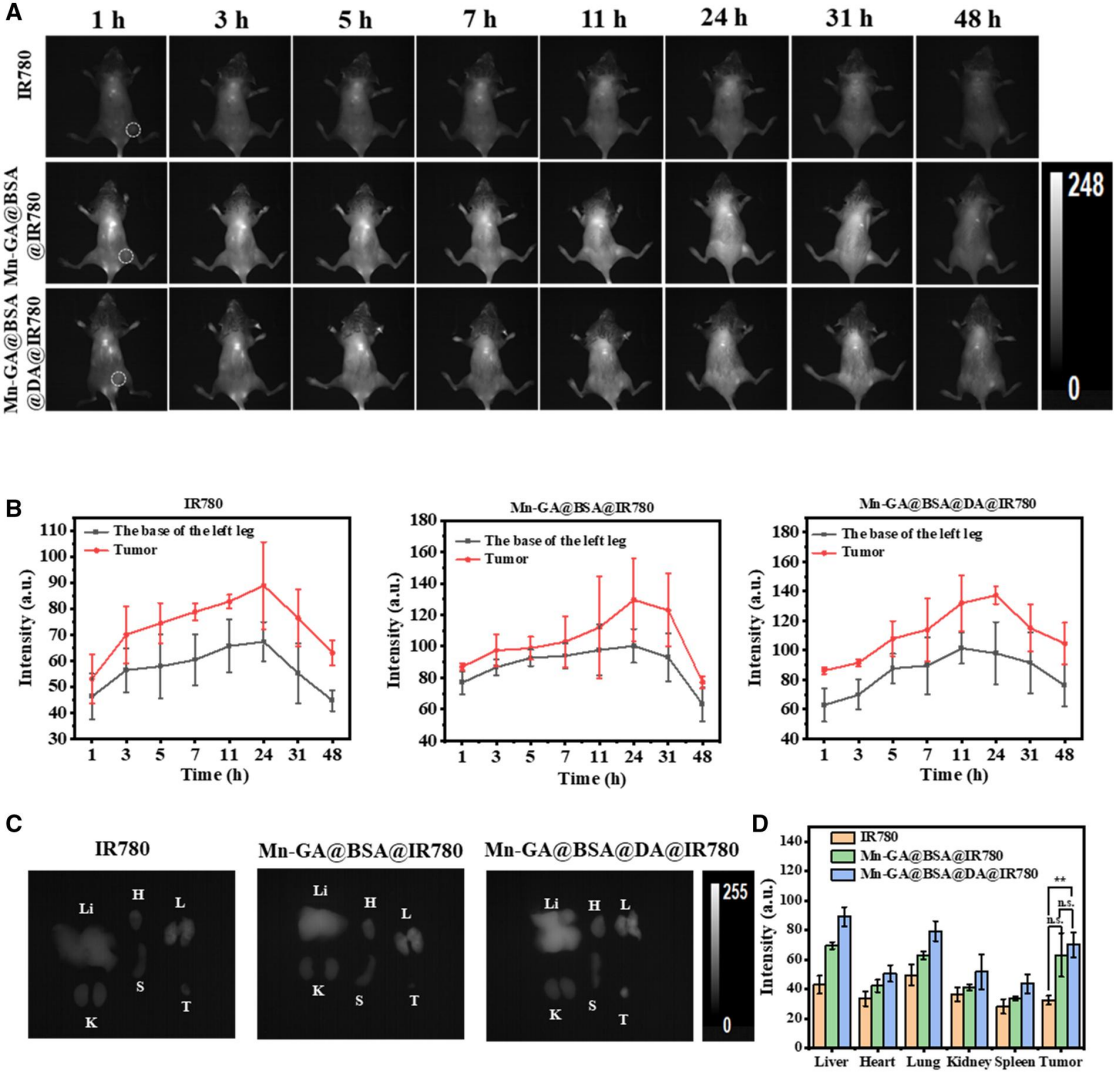
(**A**) *In vivo* fluorescence distribution of IR780, Mn-GA@BSA@IR780 and Mn-GA@BSA@DA@IR780 in tumor-bearing mice at various times points (1, 3, 5, 7, 11, 24, 31, 48 h). (**B**) Temporal changes in fluorescence signals at the tumor site and a corresponding site on the left (*n*=3). (**C** and **D**) Fluorescence image of contrast agents in the harvested normal organs and tumor tissue after 48 h post-injection (Li: liver, H: heart, L: lung, K: kidney, S: spleen, T: tumor) and the statistical analysis of the signal (*n*=3, **P<0.05).

As shown in Figure 6A, significant NIR-II signals were detected in the upper abdominal and thoracic regions of mice from both Mn-GA@BSA@IR780 and Mn-GA@BSA@DA@IR780 nanoparticle groups as early as 1 h after injection, and it also showed pronounced signals at the tumor site. The fluorescence intensity in these regions gradually increased from 1 to 11 h post-injection. Some literatures suggest that manganese complexes with lipophilic functional groups (e.g. phenyl, alkyl, benzyl, etc.) facilitate liver metabolization [53–55]; additionally, serum albumin-based nanovehicles could be metabolized by the kidney through the protein reabsorption in proximal renal tubules, mediated by Megalin/gp330 pathway [56]. Following tail vein injection, both Mn-GA@BSA@IR780 and Mn-GA@BSA@DA@IR780 exhibited accumulation in liver and kidneys, as evidenced by bright fluorescence signals.

Furthermore, the nanoparticles with a size <100 nm and coated with albumin could effectively retained in tumors through the enhanced permeability and retention effect [57, 58]. Consequently, as depicted in Figure 6A and B, the fluorescence signal at the tumor site from the Mn-GA@BSA@DA@IR780 group became increasingly pronounced 1 h post-injection. In contrast, free IR780 small molecules were quickly cleared from the bloodstream, leading to poor fluorescence tumor imaging, and at 24 h post-injection, the signal value at the tumor site in the IR780 group was only 88.8±16.80 AU, 1.71 times lower than that in the Mn-GA@BSA@DA@IR780 group. Extending the observation period to 48 h, the tumor site signal from the Mn-GA@BSA@DA@IR780 group showed the strongest fluorescence intensity at the harvested tissue about 69.95±8.43 AU (Figure 6C and D). It is primarily attributed to the high vascular permeability, and poor lymphatic drainage in tumor tissues, which slow the clearance of nanoparticles, enabling extended imaging [59]. Additionally, albumin internalization by tumor cells is mediated by receptors such as secreted protein acidic cysteine and the 60 kDa glycoprotein receptor, which are overexpressed in tumor tissue [60, 61]. And the cellular uptake of BSA-based drugs relies on the receptor-mediated endocytosis [62, 63]. Thus, this receptor-mediated mechanism enhances the uptake of albumin-based nanoparticles by tumor cells, further supporting the Mn-GA@BSA@DA contrast agent’s strong tumor-targeting imaging capabilities in vivo [64, 65].

### *T*_1_-MR imaging performance of Mn-GA@BSA@DA contrast agent *in vivo*

After evaluating the metabolic behavior of our Mn-GA@BSA@DA contrast agent, we proceeded to assess its MRI performance using a 3.0 T clinical instrument. Herein, the Mn-based contrast agents were compared with the Gd-DTPA, in tumor-bearing mice, with signal-to-noise ratio (SNR) analyzed at various time points.

**Figure 7.**
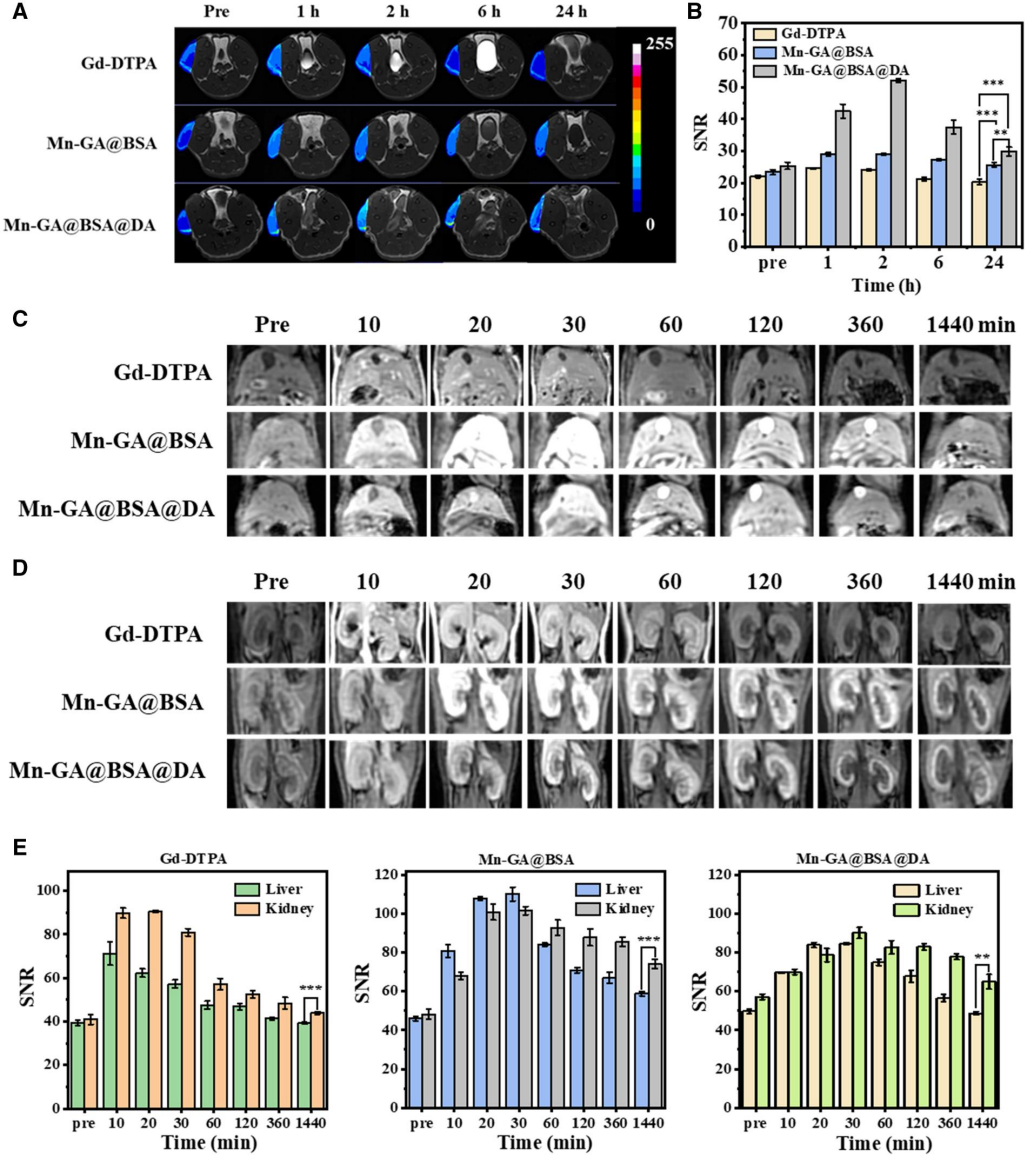
(**A**) MRI-*T*_1_-weighted imaging of subcutaneous breast cancer tumors at various time points (0, 1, 2, 6, 24 h) for Gd-DTPA, Mn-GA@BSA and Mn-GA@BSA@DA contrast agents respectively; (**B**) statistical analysis of MRI intensity in tumor regions (*n*=3). MRI-*T*_1_-weighted imaging of mice injected with Gd-DTPA, Mn-GA@BSA and Mn-GA@BSA@DA at different time points (0, 10, 20, 30, 60, 120, 360, 1440 min) for (**C**) liver and (**D**) kidney, respectively; (**E**) statistical analysis of MRI signal values in liver and kidney regions (*n*=3, **P<0.05, ***P<0.005).

As shown in Figure 7A and B, 2 h post-injection, the Mn-GA@BSA@DA agents exhibited a significant *T*_1_ signal enhancement within the tumor, with an intensity of 51.99, which was 2.05 times higher than the pre-injection value and 1.79 times greater than that of the Mn-GA@BSA particles. Notably, the clinical Gd-DTPA group displayed less than half the signal intensity of Mn-GA@BSA@DA. Moreover, while the Gd-DTPA signal returned to baseline after 6 h, Mn-GA@BSA@DA contrast agents maintained enhanced MRI in the tumor even 24 h post-injection (compared to the initial value, *P*<0.05). The control group Mn-GA@BSA showed a weak imaging effect, likely due to its poor serum stability and rapid clearance by the reticuloendothelial system, resulting in lower tumor accumulation. These excellent MRI performances of Mn-GA@BSA@DA show superior stability, prolonged circulation time and high tumor accumulation and highlights its potential as clinical contrast agent.

Interestingly, we also found imaging effects of Mn-GA@BSA@DA in the liver and kidneys of normal mice. As illustrated in Figure 7C–E, 10 min’ post-injection, the liver region of the Mn-GA@BSA@DA group exhibited significant signal enhancement from 49.67 to 69.61. Over the next 20 min, this signal showed a 1.72-fold increase. On the contrary, the Gd-DTPA signal was gradually diminished. Surprisingly, the Mn-GA@BSA control one demonstrated slightly better liver *T*_1_ imaging than the test group, with the liver *T*_1_ signal reaching 110.13 at 30 min post-injection. This difference may be related to the structural instability and increased size of Mn-GA@BSA particles in serum, making them more susceptible to phagocytosis by liver cells.

At 60 min post-injection, both the Mn-GA@BSA@DA and control Mn-GA@BSA groups displayed high brightness in the gallbladder region of the liver, indicating that the particles could transported into the bile duct and biliary system for excretion. This process resulted in a significant *T*_1_ enhancement effect in the biliary region, suggesting that, unlike the metabolism of traditional Gd-DTPA small molecules, the hepatobiliary pathway is a major metabolic route for Mn-GA@BSA@DA contrast agent metabolism.

In the renal imaging, as depicted in Figure 7D and E, the Mn-GA@BSA@DA group displayed a notable increase in kidney *T*_1_ signal just 10 min after injection, with a peak at 30 min (peak value 90.13). And the Mn-GA@BSA group showed a similar result. In contrast, the Gd-DTPA group exhibited a decline, with the *T*_1_ signal value dropping 10% within 10 min; and the signal returned to baseline levels after 24 h, indicating rapid metabolism. Both two nano-contrast agent groups maintained *T*_1_ enhancement, with a distinct ring-like effect observed in the renal cortex. This phenomenon is likely due to the BSA coating on the particles, enabling endocytosis by the renal tubules and facilitating glomerular filtration and reabsorption of protein materials [66]. Consequently, the surface modification of Mn-GA@BSA@DA particles enhanced targeting capability for kidney imaging. Even 6 h post-injection, the Mn-GA@BSA@DA group continued to show pronounced *T*_1_ enhancement in both the liver and kidneys, further confirming the prolonged MRI-*T*_1_ enhancement capabilities.

### Biocompatibility analysis of Mn-GA@BSA@DA contrast agents *in vivo*

**Figure 8.**
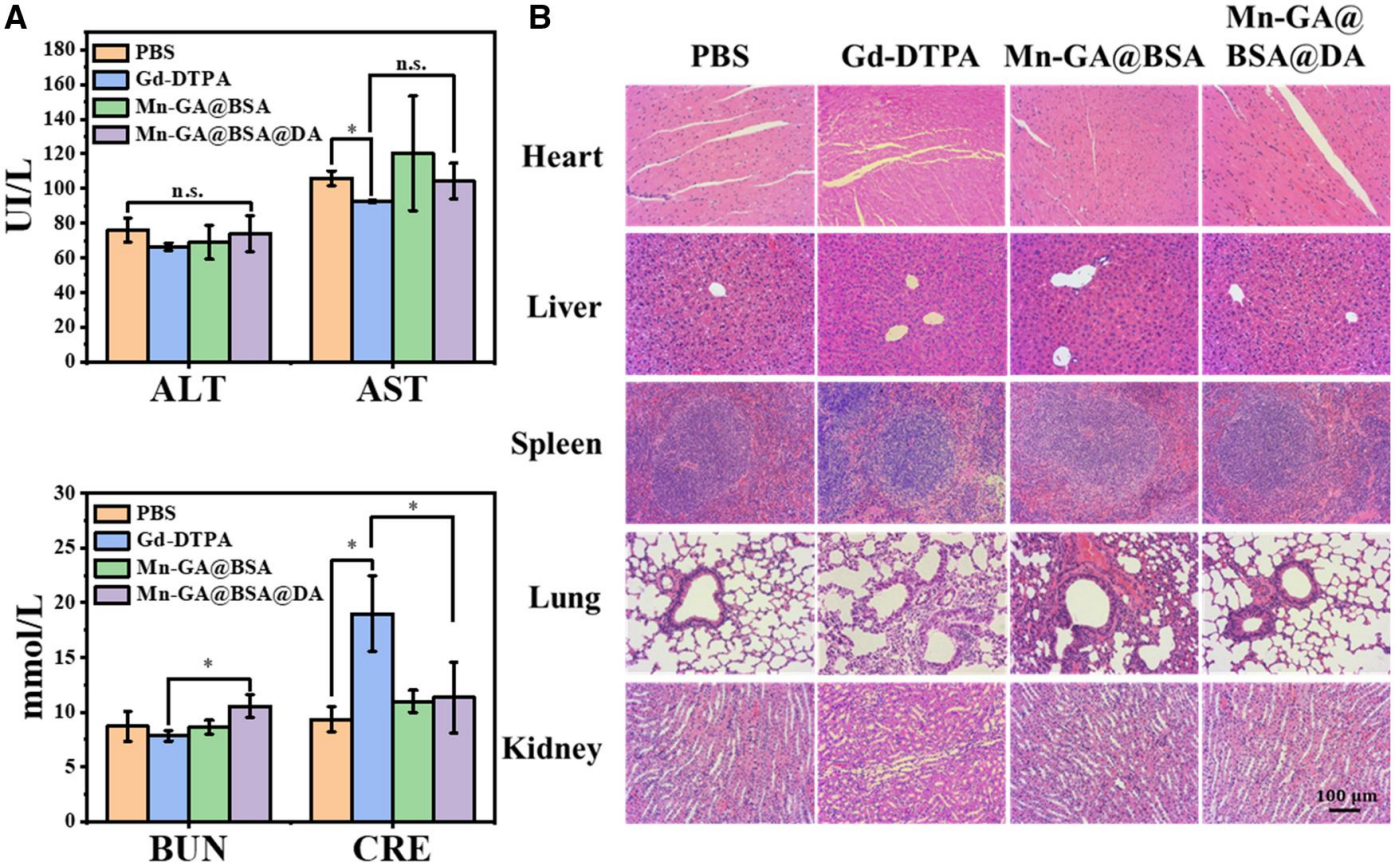
(**A**) Analysis of alanine aminotransferase (ALT), aspartate aminotransferase (AST), blood urea nitrogen (BUN) and creatinine (CRE) levels in the blood sample from PBS, Gd-DTPA, Mn-GA@BSA and Mn-GA@BSA@DA groups, respectively (*n*=3); (**B**) hematoxylin and eosin (HE) staining of major organs (heart, liver, spleen, lungs, kidneys) in mice from PBS, Gd-DTPA, Mn-GA@BSA and Mn-GA@BSA@DA groups, respectively, scale bar = 100 µm.

The specific uptake of manganese by hepatocellular and the reabsorption of protein by renal tubules contribute to the extended retention time of Mn-GA@BSA@DA contrast agents *in vivo*, necessitating a thorough assessment of their biocompatibility. To evaluate the potential toxicity of Mn-GA@BSA@DA contrast agents, we conducted a series of tests on mice. Firstly, the blood samples were collected and the analysis revealed that levels of alanine aminotransferase (ALT), aspartate aminotransferase (AST), blood urea nitrogen (BUN) and creatinine (CRE) remained within physiological norms (Figure 8A). On the contrary, it was found that the CRE value of the Gd-DTPA group was high, indicating that the application of Gd-DTPA induced a damage to renal function, which was consistent with the previous report [67]. Furthermore, we performed HE staining on tissue sections from the heart, liver, spleen, lungs and kidneys after 48 h drug injection. As illustrated in Figure 8B, the Mn-GA@BSA group showed significant inflammatory reactions in the lungs, indicating that Mn-GA@BSA contrast agents was unstable and more likely to decompose, resulting in a release of Mn^2+^, which leaded an inflammatory response in the lung tissue [68, 69]. Compared with the phosphate buffered saline (PBS) control group, Gd-DTPA also showed slight pulmonary inflammation; but in the Mn-GA@BSA@DA group, no significant pathological abnormalities were observed, demonstrating excellent structural stability and tissue compatibility.

## Conclusion

In summary, to address the limitation of clinical Gd-based contrast agents, we developed Mn-GA@BSA@DA nano-contrast agent using a fast nanoprecipitation technique. These agents were constructed through the *in situ* polymerization of DA with albumin, followed by cross-linking with Mn^2+^. This FNP technique used here facilitates the large-scale, cost-effective production of these contrast agents, meeting industrial demands while simplifying the preparation process. Under optimized synthesis conditions, this manganese-based contrast system exhibited a hydrodynamic size of approximately 18 nm, excellent stability in physiological environments and outstanding *T*_1_-weighted imaging performance with high *r*_1_ value (18.5 mM^−1^ s^−1^ at 3.0 T) and a low *r*_2_/*r*_1_ ratio (3.78). *In vivo* studied showed Mn-GA@BSA@DA nanoparticles surpassed Gd-DTPA in tumor MRI diagnostic efficacy, while also offering an extended imaging window. This contrast agent was metabolized and excreted through hepatic and renal metabolic pathways and showed high biosafety profile and significant potential as a clinical contrast agent. Furthermore, this method for preparing Mn-GA@BSA@DA nano-contrast agent holds promise for further extension to other proteins or self-assembled systems, and this work provides a versatile strategy for developing next-generation MRI contrast agents.

## Supplementary Material

rbaf009_Supplementary_Data

## Data Availability

Data will be made available on request.
