# Supplementary material for: Facile construction of manganese-based contrast agent with high T1 relaxivity for magnetic resonance imaging via flash technology-based self-assembly
Source: Regen Biomater. 2025 Mar 11;12:rbaf009. doi: 10.1093/rb/rbaf009 (PMC12017619; doi:10.1093/rb/rbaf009)
Supplement: rbaf009_Supplementary_Data [file rbaf009_supplementary_data.zip › Supplementary Material.docx]

**Supporting Information**

**Facile Construction of Manganese-based Contrast Agent with High *T*_1_ Relaxivity for Magnetic Resonance Imaging via Flash Technology-based Self-assembly**

Chunwei Wu^1^$\dagger$, Jie Zhong^2^$\dagger$, Jianing Li^4^, Yande Luo^5^, Junyao Wang^1^, Xiaodie Zeng^1^, Jiaji Mao^4^, Jianping Lu^1^, Junyao Xu^3^*, Changqiang Wu^2^*, ^2^*and Zhiyong Wang^1^*

1. School of Materials Science and Engineering, Center for Functional Biomaterials, Key Laboratory for Polymeric Composite and Functional Materials of Ministry of Education, Sun Yat-sen University, Guangzhou 510275, P. R. China
2. Medical Imaging Key Laboratory of Sichuan Province and School of Medical Imaging, North Sichuan Medical College, Nanchong 637000, P. R. China
3. The Third affiliated hospital, Guangzhou Medical University, Guangzhou 510150, P. R. China
4. Department of Radiology, Guangdong Provincial Key Laboratory of Malignant Tumor Epigenetics and Gene Regulation, Sun Yat-Sen Memorial Hospital, Sun Yat-Sen University, Guangzhou 510120, P. R. China
5. School of Biomedical Engineering， Guangzhou Medical University，Guangzhou 511495, P. R. China

*Correspondence address.

E-mail: wangzhiy3@mail.sysu.edu.cn (Z.Y. Wang)

wucq1984@nsmc.edu.cn (C.Q. Wu) ;

2023683035@gzhmu.edu.cn(J.Y. Xu) ;

$\dagger$These authors contributed equally to this work.


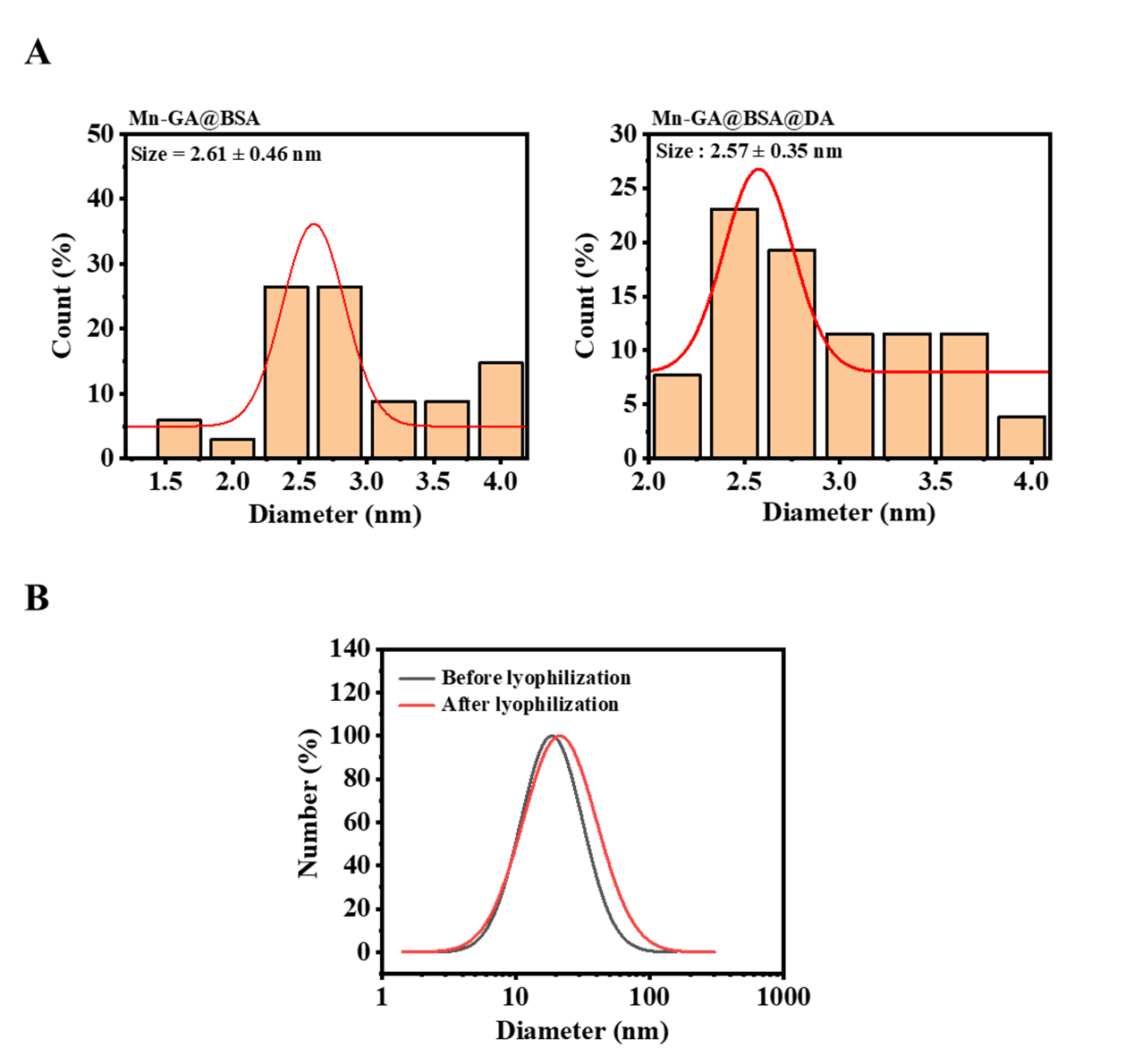


**Figure S1.** (A) TEM analysis of the Mn-GA@BSA and Mn-GA@BSA@DA particles’ size distribution, respectively; (B) Dynamic size analysis of Mn-GA@BSA@DA before and after lyophilization.


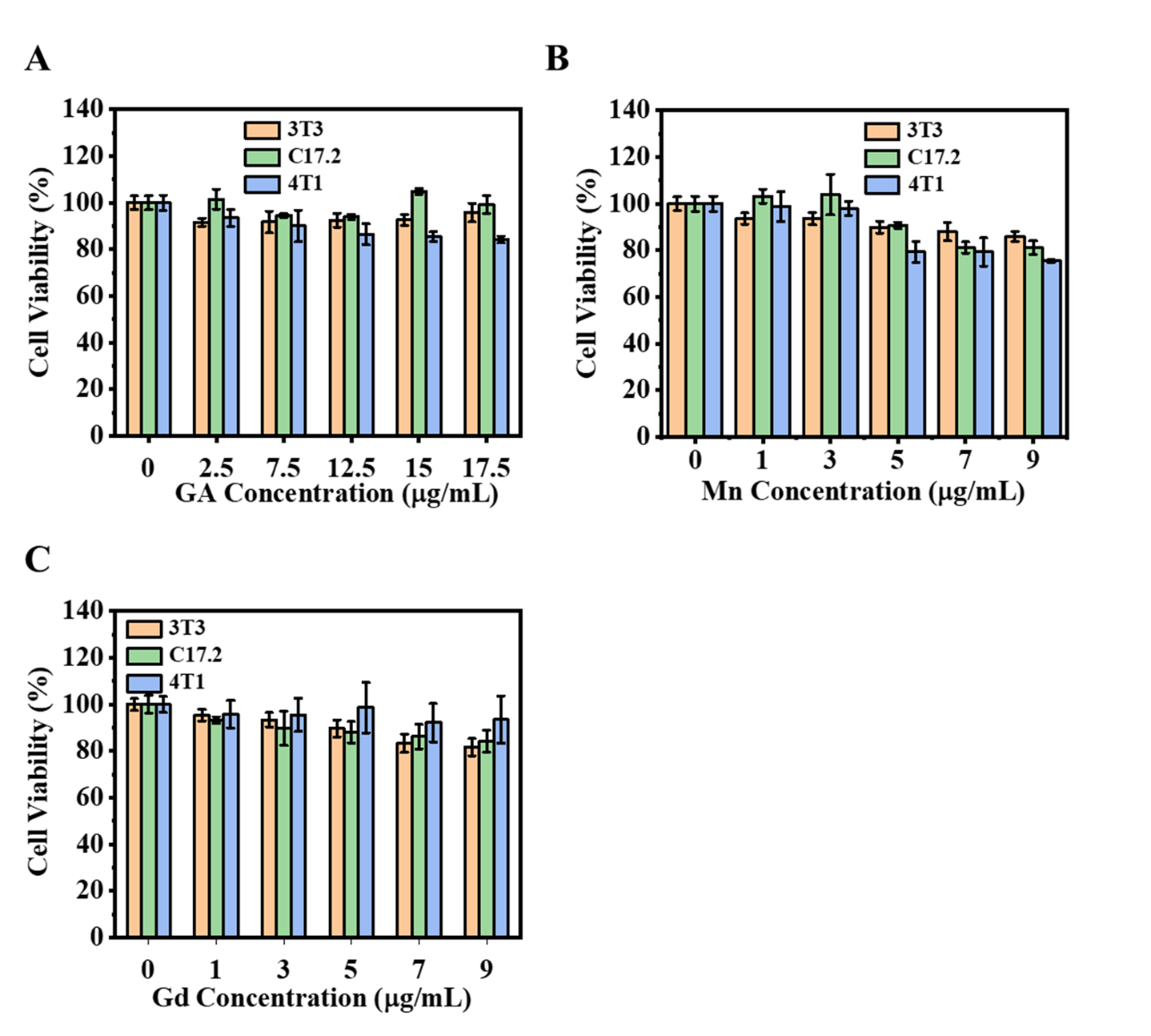


**Figure S2.** Cytotoxicity analysis of (A) pyrogallic acid, (B) MnCl_2_ and (C) Gd-DTPA on 3T3, C17.2 and 4T1 cells, respectively (n=3).


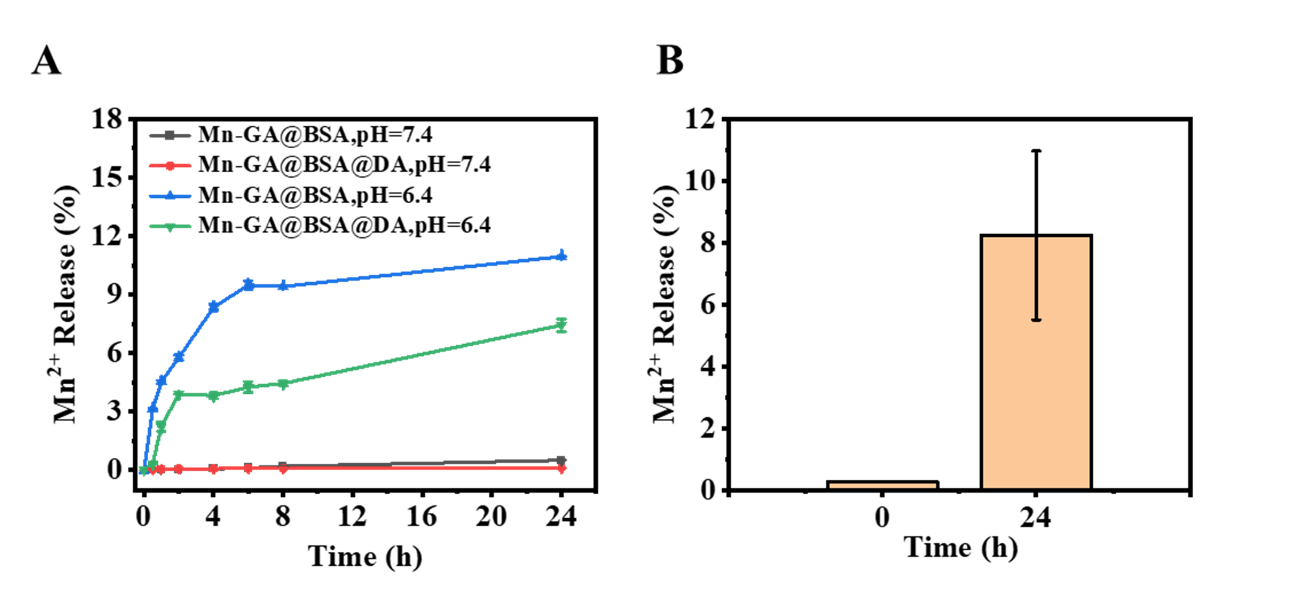


**Figure S3.** release profiles of Mn^2+^ from (A). Mn-GA@BSA and Mn-GA@BSA@DA over times (0, 0.5, 1, 2, 4, 6, 8, 24 h) under physiological environment at pH 7.4 and 6.4; (B). Mn-GA@BSA@DA at times (0, 24 h) within 40 times dosage of Zn^2+^ at pH 6.4.(n=3)


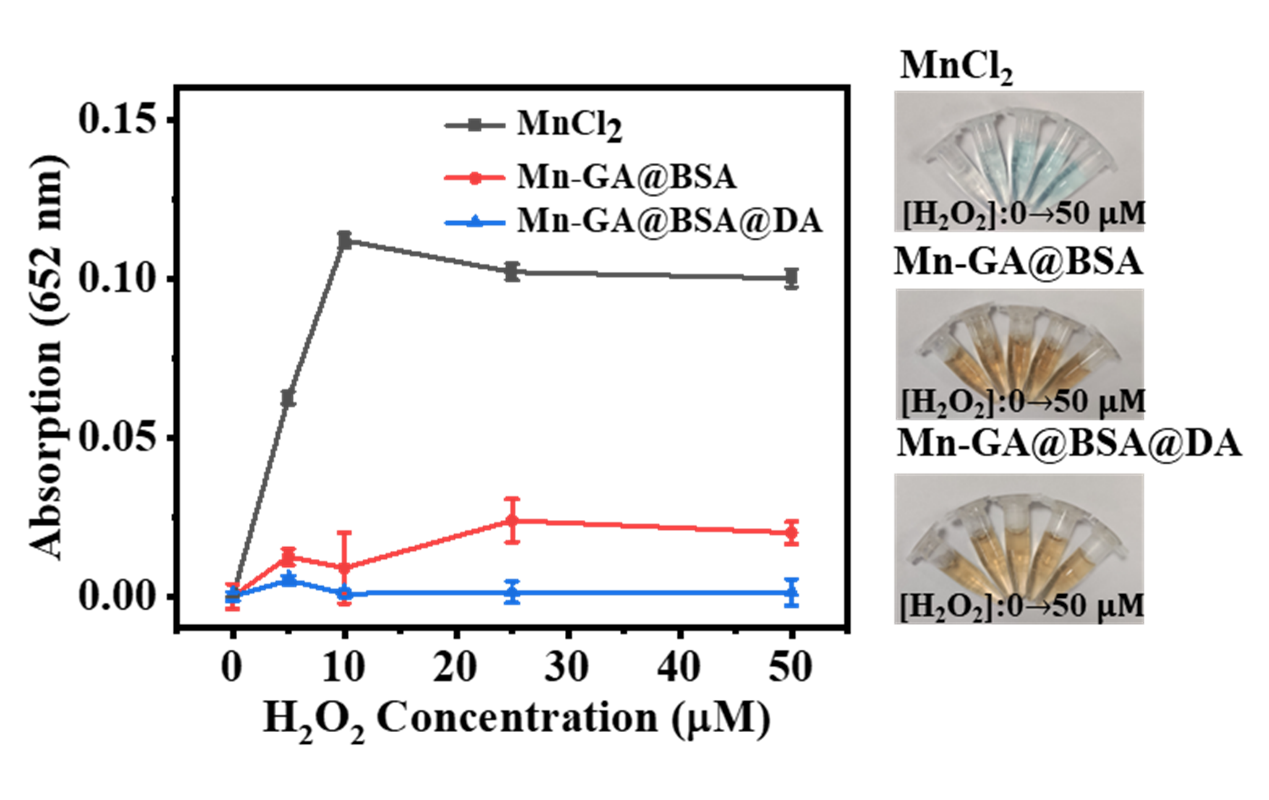


**Figure S4.** Color changes in TMB during the Fenton reaction with MnCl_2_, Mn-GA@BSA and Mn-GA@BSA@DA agents at varying concentrations of H_2_O_2_, along with corresponding changes in absorption values at 652 nm. (n=3)


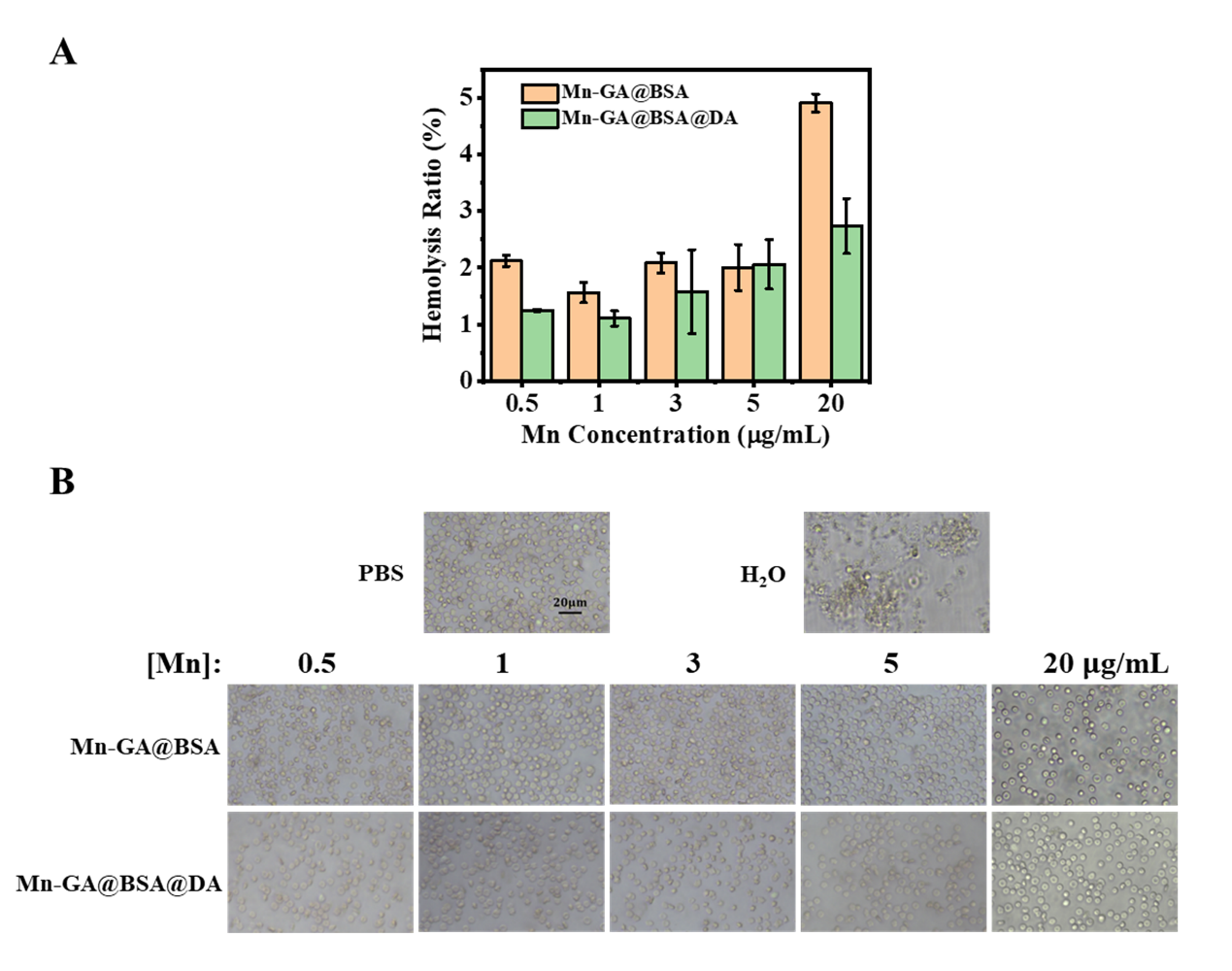


**Figure S5.** (A) Hemolysis assays of Mn-GA@BSA and Mn-GA@BSA@DA nanoparticles at various concentrations (n=3); (B) White light images of erythrocytes under an inverted microscope corresponding to different concentration, with a scale bar of 20$\mu m$.


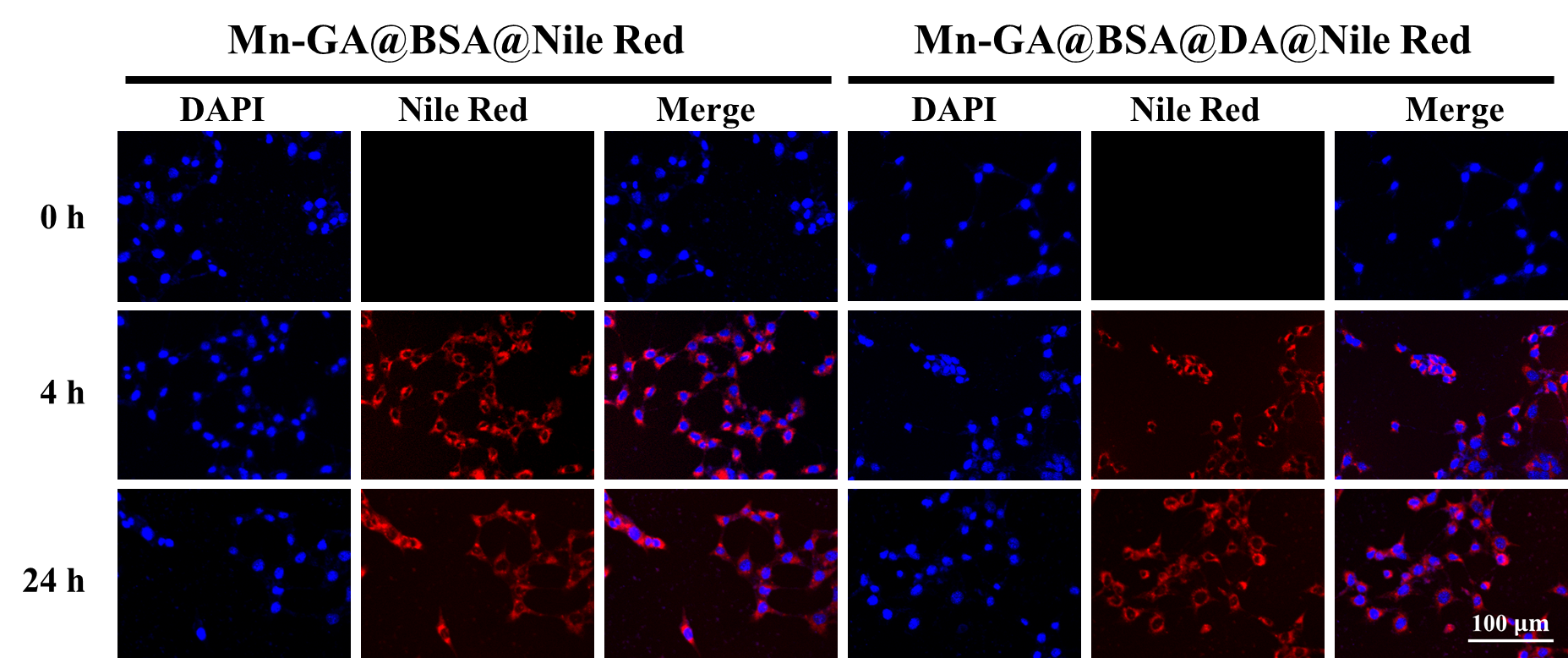


**Figure S6.** The cell uptake results of Mn-GA@BSA@Nile Red and Mn-GA@BSA@DA@Nile Red nanoparticles by 4T1 cells at different time points (0, 4, 24 h) were measure through confocal scanning laser microscope, bar = 100 μm.

**Figure S7.** Cell uptake of Gd-DTPA, Mn-GA@BSA and Mn-GA@BSA@DA contrast agents at different time points (0.25, 4, 24 h) under the same metal concentration (n=3, **P<0.05, ***P<0.005)


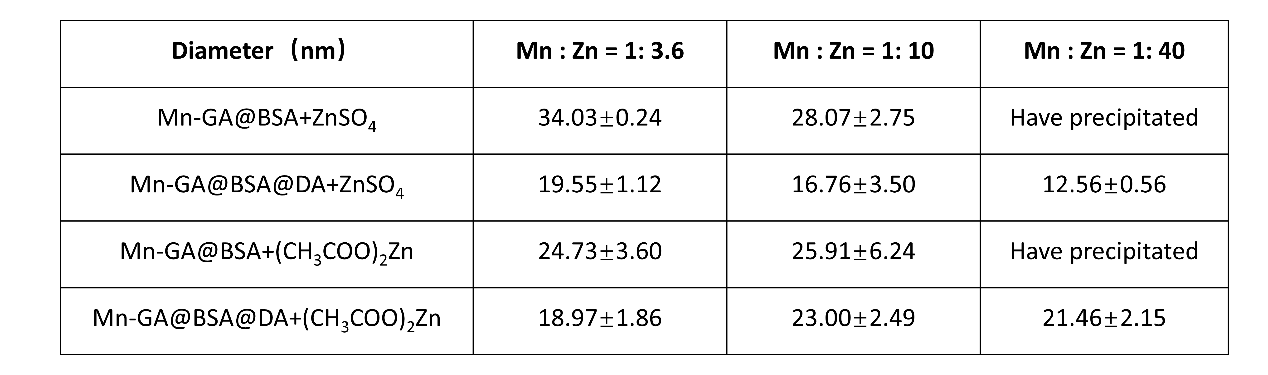


**Table 1**. Particle sizes of Mn-GA@BSA and Mn-GA@BSA@DA contrast agents in ZnSO_4_ and (CH_3_COO)_2_Zn solutions with Mn: Zn ratios of 1: 3.6, 1: 10 and 1: 40, respectively.
